# Preservation vs. Resection? Pediatric and Non-Pediatric Management Patterns in Ovarian Torsion

**DOI:** 10.3390/pediatric18020032

**Published:** 2026-03-02

**Authors:** Xiaoyan Feng, Peter Zimmermann, Nicolas Pardey, Richard Gnatzy, Stefan Bassler, Jona T. Stahmeyer, Martin Lacher, Jan Zeidler

**Affiliations:** 1 Department of Pediatric Surgery, University of Leipzig, 04103 Leipzig, Germany; xiaoyan.feng@medizin.uni-leipzig.de (X.F.); richard.gnatzy@medizin.uni-leipzig.de (R.G.);; 2 Department of Pediatric Surgery and Congenital Malformations, Helios Dr. Horst Schmidt Kliniken Wiesbaden, 65199 Wiesbaden, Germany; 3 Center for Health Economics Research Hannover (CHERH), 30159 Hannover, Germany; nicolas.pardey@like-healthcare.de (N.P.); jz@cherh.de (J.Z.); 4 AOK PLUS-Die Gesundheitskasse fuer Sachsen und Thueringen, 01067 Dresden, Germany; 5 AOK-Die Gesundheitskasse fuer Niedersachsen, 30519 Hannover, Germany; jona.stahmeyer@nds.aok.de

**Keywords:** ovarian torsion, department, university/maximum care, treatment

## Abstract

Background: Ovarian torsion (OT) is a rare but urgent surgical condition in children and adolescents. Evidence on how management differs between pediatric (PD) and non-pediatric (Non-PD) departments in Germany remains limited. Methods: We conducted a retrospective cohort study using anonymized claims data from two major German statutory health insurance funds (2010–2019), covering 6.3 million insured individuals (≈1 million children). Patients ≤18 years with an inpatient diagnosis of OT (ICD-10-GM N83.5) were analyzed with respect to demographics, department type (PD vs. Non-PD), hospital type (university/maximum care [UM] vs. non-university/maximum care [Non-UM]), surgical procedures, and outcomes. Results: A total of 293 patients (mean age 12.4 ± 4.5 years) were included; 71% were adolescents (12–18 years). Adolescents were predominantly treated in Non-PD (89%), whereas younger children were more often managed in PD (50%; *p* < 0.0001). Most cases were treated in Non-UM (82%). Laparoscopy was more commonly used in Non-PD departments (85%), while open surgery and oophorectomy occurred more frequently in PD and university hospitals (UM). Ovary-sparing procedures accounted for 77% of all cases, whereas 23% underwent oophorectomy. Mean hospital stay was longer in PD (6.7 ± 9.0 days) than in Non-PD (4.9 ± 2.2 days; *p* = 0.0167). Readmission rates were comparable across groups. Conclusions: Management of OT in Germany varies markedly by department and hospital type. PD and UM treat more younger patients but perform oophorectomy more frequently, whereas Non-PD and Non-UM favor laparoscopic, ovary-sparing strategies. These differences highlight the urgent need for standardized, evidence-based protocols prioritizing ovarian preservation and optimizing long-term outcomes in affected children and adolescents.

## 1. Introduction

Ovarian torsion (OT) is a surgical emergency, responsible for approximately 2–7% of acute abdominal cases in females [1,2]. The incidence of OT in the pediatric population is estimated to range from 4.9 per 100,000 to 20–30 per 100,000, with an average age of onset between 13 and 14.5 years [3,4]. The most common clinical presentation of OT is sudden onset of localized lower abdominal pain with or without nausea and vomiting. Imaging techniques, such as ultrasonography, computed tomography (CT), magnetic resonance imaging (MRI), and others, may assist in the diagnosis of OT. However, these imaging modalities cannot definitively confirm or rule out the condition [5,6]. To prevent ovarian necrosis and severe complications, immediate exploratory laparoscopy is recommended when symptoms strongly indicate ovarian torsion.

Surgical management options include detorsion alone, detorsion with oophoropexy, ovarian cystectomy, and oophorectomy, performed either via open or laparoscopic approaches. Practice patterns vary between pediatric and non-pediatric surgeons, as well as between teaching and non-teaching hospitals [7]. Notably, recent studies have found no clear evidence supporting the benefit of oophorectomy in managing ovarian torsion (OT) in pediatric patients [8,9]. In contrast, most salvaged ovaries regain viability and function following detorsion, regardless of their intraoperative appearance. Significant differences in the treatment of ovarian torsion (OT) between pediatric and non-pediatric departments reveal a clear research gap. Further investigation is needed to identify the optimal treatment strategy that ensures ovarian preservation while minimizing surgical intervention. This observational cohort study aims to assess the management of children and adolescents with OT in both pediatric (PD) and non-pediatric (Non-PD) departments in Germany.

We hypothesize that management strategies for OT in this population vary significantly depending on department type (PD vs. Non-PD). Additionally, these differences are influenced by hospital type (university/maximum care hospitals “UM” vs. non-university/maximum care hospitals “Non-UM”), with pediatric departments likely employing more advanced approaches.

## 2. Methods

This retrospective cohort study used anonymized claims data from two major German statutory health insurance providers, covering the period from 2010 to 2019. The dataset comprises approximately 6.3 million insured individuals, including roughly 1 million children and adolescents, offering a robust base for examining rare pediatric conditions such as ovarian torsion.

Patients aged 18 years or younger with an inpatient diagnosis of ovarian torsion were identified using ICD-10-GM code N83.5. To ensure adequate follow-up, only those with continuous insurance coverage for at least one year after the index hospitalization (or until death) were included. No malignant gynecologic or extragonadal pelvic tumors (ICD-10 C56, C57, C76.2, C76.3) were detected in the dataset.

Variables included age category, department type (pediatric or non-pediatric), hospital type (university/maximum care or non-university/maximum care), surgical approach (laparoscopic or open), length of stay and readmission within one year. Operative procedures were categorized using ICPM codes. Following reviewer recommendations, operative management was reorganized into two overarching categories: oophorectomy (open or laparoscopic) and ovary-sparing procedures, which were further subdivided into detorsion only and detorsion with adjunctive procedures such as cystectomy, fenestration, wedge excision or parovarian cyst excision. Single-incision laparoscopy was regarded as an operative approach rather than a distinct procedure, while adhesiolysis was considered unrelated to torsion management and oophoropexy was classified as a recurrence-prevention measure.

Categorical variables were analyzed using the chi-square or Fisher’s exact test, and continuous variables using the Mann–Whitney U test. Variance homogeneity and multicollinearity were assessed using the Levene test and variance inflation factors. Statistical significance was defined as *p* < 0.05. Analyses were performed using SAS 9.4 (SAS Institute, Cary, NC, USA).

The study complied with Good Practice Secondary Data Analysis guidelines. Ethical approval was granted by the local institutional review board (IRB 022/21-ek). Because the data were fully anonymized and derived from routine claims, informed consent was not required. All analyses conformed to the Declaration of Helsinki and the GDPR.

## 3. Results

### 3.1. Patient Characteristics

A total of 293 children and adolescents diagnosed with ovarian torsion (ICD-10-GM code N83.5) were included in the study. The mean age was 12.4 ± 4.5 years. Of these, 209 (71%) were adolescents aged 12 years or older, while 84 (29%) were children under 12 years of age. Adolescents were predominantly treated in non-pediatric departments (Non-PD: 89%; PD: 50%; *p* < 0.0001), whereas younger children (<12 years) were more frequently admitted to pediatric departments (PD: 50%; Non-PD: 11%; *p* < 0.0001). Most patients were treated in non-university hospitals (Non-UM: 82%) compared to university hospitals (UM: 18%; *p* < 0.0001) (Main Table 1).

### 3.2. Use of Laparoscopy and Operative Approaches

A total of 330 surgical procedures encompassing 15 different techniques were performed. Laparoscopic surgery was significantly more frequent in non-pediatric departments (85.3%) than in pediatric departments (63%; *p* = 0.0012) (Table 1). No significant differences were found in laparoscopy rates between university hospitals (64%) and non-university hospitals (79%; *p* = 0.8688) (Table 1). Single incision laparoscopic surgery (SILS) was rarely performed, accounting for only 3 cases (2%) in pediatric departments and none (0%) in non-pediatric departments (*p* = 0.0779) (Supplement Table S1).

The predominant procedure was laparoscopic ovarian detorsion (21%), with a small proportion performed as open or unspecified detorsions (1%) (Supplement Tables S1 and S2). Ovarian cyst fenestration/cystectomy was performed laparoscopically in 27% of cases, with significantly higher rates in non-pediatric departments (36%) compared to pediatric departments (16%; *p* = 0.0003) (Supplement Table S1). Open or unspecified excisions/incisions were rare (2%) and did not differ significantly between groups (Supplement Table S2).

Oophorectomy was performed in a subset of cases, with both laparoscopic and open approaches utilized. Laparoscopic oophorectomy or salpingo-oophorectomy was performed in 9% of cases, with similar rates observed in pediatric departments (11%) and non-pediatric departments (7%; *p* = 0.2701) (Supplement Table S1). Open or unspecified oophorectomy was performed in 14% of cases, being significantly more common in pediatric departments (26%) than non-pediatric departments (4%; *p* < 0.0001) (Supplement Table S1). Similar trends were observed between university and non-university hospitals, where laparoscopic oophorectomy was less frequent (4% vs. 10%; *p* = 0.2559) compared to open or unspecified procedures (28% vs. 10%; *p* = 0.0011) (Supplement Table S2).

When comparing PD and Non-PD departments, open oophorectomy was significantly more frequent in pediatric departments (26%) compared to non-pediatric departments (4%; *p* < 0.0001). In contrast, laparoscopic oophorectomy rates were similar between PD (11%) and Non-PD (7%; *p* = 0.2701).

Among university (UM) and non-university (Non-UM) hospitals, open oophorectomy was performed at a higher rate in university hospitals (28%) than in non-university hospitals (10%; *p* = 0.0011). Laparoscopic oophorectomy, however, was less frequent in UM (4%) compared to Non-UM hospitals (10%; *p* = 0.2559).

Additional laparoscopic procedures included excision of parovarian cysts (6%) and wedge excisions (9%), both performed more frequently in non-pediatric and non-university hospitals (Supplement Tables S1 and S2). Open approaches for these procedures were rare (<1%). Totally 81 operation and procedure codes (OPS) combinations were identified, which revealed that the top 3 combinations accounted for 48% of cases, with the most common being laparoscopic detorsion combined with cystectomy (28%) (Supplement Table S3). Rare procedure combinations made up 20% of cases, emphasizing the need for individualized and specialized surgical approaches (Supplement Table S3).

### 3.3. Clinical Outcomes

No patients had comorbidities, including malignant neoplasms (ICD-10-GM codes: C56, C57, C76.2, C76.3). The overall mean hospital stay was 5.7 ± 6.3 days, with patients in pediatric departments experiencing significantly longer stays (6.7 ± 9.0 days) compared to those in non-pediatric departments (4.9 ± 2.2 days; *p* = 0.0167). However, hospital stay duration did not differ significantly between university (6.0 ± 8.3 days) and non-university hospitals (5.9 ± 5.9 days; *p* = 0.4511). Readmission rates were comparable between PD (14%) and Non-PD (20%; *p* = 0.1823) as well as between UM (17%) and Non-UM (17%; *p* = 0.9836), suggesting consistent long-term outcomes regardless of department or hospital type (Table 1). No malignant tumors or chemotherapy codes were identified. Recurrent torsion within one year was extremely rare (<2%).

## 4. Discussion

This observational cohort study examined the management of children and adolescents with OT (ovarian torsion) in PD (pediatric department) and Non-PD, as well as in UM (university/maximum care hospitals) and Non-UM in Germany. Data were sourced from two major public health insurance providers in Germany, covering 6.3 million insured individuals, including approximately 1 million children, providing a representative patient sample. Previous studies by our group have demonstrated that claims data are a reliable source of evidence for evaluating pediatric healthcare services.

### 4.1. Central Role of Pediatric and University Hospitals in Younger OT Patients

OT cases were nearly equally distributed between PD and Non-PD; however, PDs treated a significantly higher proportion of children under 12 years of age. Adolescents were predominantly managed in Non-PD settings (89%), reflecting referral patterns and departmental specialization. Similarly, UM hospitals treated a significantly higher proportion of younger children (46% vs. 25% in Non-UM), reinforcing their focus on younger pediatric patients. These findings confirm that PD and UM institutions are central providers for younger children.

### 4.2. Ovary Preservation vs. Resection: Departmental and Institutional Differences

Non-PD departments performed significantly more ovary-sparing cystectomy/fenestration procedures, and fewer open oophorectomies. Similar trends have been reported in the United States, where up to 38% of pediatric patients with OT underwent oophorectomy when treated by pediatric surgeons, whereas gynecologists demonstrated a stronger preference for ovary-sparing techniques [10]. With increasing awareness of the importance of preserving ovarian function, numerous studies have emphasized ovary-preserving surgery, which has been shown to maintain reproductive potential in females with adnexal torsion [11,12].

In addition, we found that UM hospitals manage a higher proportion of pediatric-specific and potentially more complex OT cases compared to Non-UM institutions. Although they represent only 1.7% of hospitals in Germany, UMs treated 18% of all OT cases—highlighting their role as centers for specialized care. Moreover, UMs performed more open oophorectomies (28%) compared to Non-UM hospitals (10%). However, due to the lack of detailed clinical data on case severity, intraoperative findings, and patient comorbidities, it remains unclear whether the observed differences in treatment approaches truly reflect higher case complexity or institutional surgical practices. Further research incorporating prospective clinical data is necessary to validate whether UM systematically manages more severe cases or simply employs different treatment strategies compared to Non-UM hospitals.

### 4.3. Adoption and Utilization of Laparoscopy in Ovarian Torsion

Overall, 76% of all procedures were performed laparoscopically. Laparoscopy rates were significantly higher in Non-PD departments. When comparing UM and Non-UM hospitals, the difference was less pronounced. The lower prevalence of laparoscopy in PD may be linked to the historical adoption of minimally invasive surgery (MIS). Nezhat et al. reported the first laparoscopic management of OT in adults as early as 1991, whereas Steyaert et al. emphasized its use in children only in 1998 [13,14]. In a bibliometric study of pediatric MIS covering three decades, our group demonstrated a delayed but subsequently rapid uptake of MIS in pediatric surgery [15]. These trends suggest that laparoscopy will likely become the national standard of care in Germany in the near future.

### 4.4. Procedural Patterns: Few Common Combinations Dominate Practice

Although 81 OPS-coded combinations were identified, nearly half of all cases were concentrated in just three: detorsion with cystectomy (28%), detorsion with wedge excision (12%), and cystectomy with parovarian cyst excision (7%). Simple single procedures (detorsion only, oophorectomy only, cystectomy only) accounted for more than one-third of cases. Rare combinations (<2 cases each) represented only 17%. Thus, while coding suggests a wide spectrum, clinical practice is dominated by a few standard approaches.

### 4.5. Low Malignancy Rates in Pediatric Ovarian Torsion

No malignant neoplasms were documented in this cohort. This is consistent with the rarity of ovarian tumors in children (annual incidence 2.2–2.6 per 100,000) and reflects the study’s selection criteria focusing on torsion (ICD-10 N83.5). Additional factors include diagnostic priorities in acute settings, coding limitations, and the absence of histopathological data in claims datasets. These constraints may have led to underreporting.

### 4.6. Clinical Outcomes Independent of Surgical Strategy

The length of hospital stay was significantly longer in PD compared to Non-PD, a pattern similarly observed in our previous studies examining appendectomy outcomes [16]. Between UM and Non-UM, LOS did not differ. Readmission rates were comparable. Thus, despite different surgical strategies, short-term outcomes appear broadly similar.

### 4.7. Limitations

Key limitations include the absence of detailed clinical data (ovarian viability, intraoperative findings, long-term fertility), potential coding inconsistencies, and lack of information on the operating surgeon’s specialty. Detailed reoperation data cannot be reliably distinguished in claims datasets unless coded as a separate hospitalization or distinct primary procedure. The dataset covers only patients insured by two large public health funds, limiting generalizability. Importantly, higher oophorectomy rates in PD and UM cannot be conclusively attributed to greater case complexity without severity markers.

### 4.8. Authors’ Experience

In the authors’ department, all patients with suspected OT undergo diagnostic laparoscopy. As pediatric surgeons at a UM, we follow a consistent strategy of detorsion and ovarian preservation with SILS technique, regardless of the intraoperative appearance of the ovary or fallopian tube, even in cases with suspected teratoma. In such situations, additional diagnostic imaging, such as MRI, is required before planning ovarian-sparing teratoma resection or oophorectomy. This strategy maximizes the chances of preserving fertility and allows for a planned “second-look” procedure during follow-up if necessary. Organ resection should always be considered a last resort.

Although transferring all patients with suspected OT to a UM with PD is not feasible, a standardized nationwide diagnostic and treatment protocol should be established to ensure optimal patient care.

## 5. Conclusions

This large-scale analysis reveals pronounced variation in the management of pediatric ovarian torsion across German institutions. Pediatric and university departments treated younger children but performed oophorectomy more frequently and relied more on open surgery, whereas non-pediatric and non-university hospitals demonstrated greater use of laparoscopy and higher rates of ovarian preservation. These differences underscore the need for national standardization, emphasizing minimally invasive detorsion and ovary preservation as the default strategy. Prospective multicenter studies that incorporate intraoperative severity markers and long-term functional outcomes are essential to optimize care and ensure uniformity across institutions.

## Figures and Tables

**Table 1:** Patient characteristics (Pediatric Departments (PD)/Non-Pediatric Departments (Non-PD); University/Maximum care hospitals (UM)/Non-University/Maximum care hospitals (Non-UM)).

Variable	All	PD	Non-PD	*p* (PD vs. Non-PD)	UM	Non-UM	*p* (UM vs. Non-UM)
Total patients [*n*]	293	134	159		52	241	
Mean age (years)	12.4 ± 4.5	10.1 ± 5.2	14.4 ± 2.5	0.0032	12.9 ± 4.2	10.3 ± 5.3	<0.0001
Children <12 y [*n*, %]	84 (29%)	67 (50%)	17 (11%)	<0.0001	24 (46%)	60 (25%)	0.0090
Adolescents 12–18 y [*n*, %]	209 (71%)	67 (50%)	142 (89%)	<0.0001	28 (54%)	181 (75%)	<0.0001
Laparoscopy [*n*, %]	251 (76%)	89 (63%)	162 (85%)	0.0012	43 (64%)	208 (79%)	0.8688
Open surgery [*n*, %]	79 (24%)	52 (37%)	27 (15%)	<0.0001	24 (36%)	55 (21%)	0.0005
Oophorectomy total [*n*, %]	75 (23%)	53 (38%)	22 (12%)	<0.0001	22 (33%)	53 (20%)	0.0022
Oophorectomy laparoscopic [*n*, %]	30 (9%)	16 (11%)	14 (7%)	0.2701	3 (5%)	27 (10%)	0.2559
Oophorectomy open [*n*, %]	45 (14%)	37 (26%)	8 (4%)	<0.0001	19 (28%)	26 (10%)	0.0011
Length of stay (days)	5.7 ± 6.3	6.7 ± 9.0	4.9 ± 2.2	0.0167	6.0 ± 8.3	5.9 ± 5.9	0.4511
Readmission [*n*, %]	51 (17%)	19 (14%)	32 (20%)	0.1823	9 (17%)	42 (17%)	0.9836

## Data Availability

The data that support the findings of this study are available from a third party, but restrictions apply to the availability of these data, which were used under license for the current study, and so are not publicly available. Data are available from the authors upon reasonable request and with permission of the third party.

## Supplementary Materials

The following supporting information can be downloaded at: https://www.mdpi.com/article/10.3390/pediatric18020032/s1, Table S1: Surgical procedures by department type (PD vs. Non-PD); Table S2: Surgical procedures by hospital type (UM vs. Non-UM); Table S3: Common distribution of the procedures.
