# Supplementary material for: Preservation vs. Resection? Pediatric and Non-Pediatric Management Patterns in Ovarian Torsion
Source: Pediatr Rep. 2026 Mar 2;18(2):32. doi: 10.3390/pediatric18020032 (PMC13010654; doi:10.3390/pediatric18020032)
Supplement: Supplementary file 1 [file pediatrrep-18-00032-s001.zip › pediatrrep-3999538-supplementary.pdf]

Supplement Table 1 Surgical procedures by department type (PD vs. Non-PD)

Type of treatment / procedure	All departments (n=330)	PD (n=141)	Non-PD (n=189)	p-value
**Detorsion (lap)**	69 (20.8%)	28 (19.9%)	41 (21.7%)	0.808
**Detorsion (open / non specified)**	4 (1.2%)	2 (1.4%)	2 (1.1%)	1.000
**Cyst fenestration / cystectomy (lap)**	90 (27.2%)	22 (15.6%)	68 (36.0%)	0.0003
**Cyst excision/incision (open / non specified)**	6 (1.8%)	4 (2.8%)	1 (0.5%)	0.412
**Oophorectomy (lap)**	30 (9.1%)	16 (11.4%)	14 (7.4%)	0.270
**Oophorectomy (open / non specified)**	45 (13.6%)	37 (26.2%)	8 (4.2%)	<0.0001
**Parovarian cyst excision (lap)**	20 (6.0%)	7 (5.0%)	13 (6.9%)	0.652
**Parovarian cyst excision (open / non specified)**	1 (0.3%)	0 (0.0%)	1 (0.5%)	1.000
**Ovariopexy (lap)**	8 (2.4%)	4 (2.8%)	4 (2.1%)	0.731
**Wedge excision / biopsy (lap)**	28 (8.5%)	9 (6.4%)	19 (10.1%)	0.340
**Wedge excision / biopsy (open / non specified)**	3 (0.9%)	2 (1.4%)	1 (0.5%)	0.579
**Miscellaneous / non specified**	19 (5.7%)	5 (3.6%)	14 (7.4%)	0.170
**Single-port laparoscopy**	3 (0.9%)	3 (2.1%)	0 (0.0%)	0.078
**Adhesiolysis (lap)**	3 (0.9%)	0 (0.0%)	3 (1.6%)	0.266
**Adhesiolysis (open / non specified)**	2 (0.6%)	2 (1.4%)	0 (0.0%)	0.182

Supplement Table 1. Surgical procedures by department type (PD vs. Non-PD): Non-PD departments performed significantly more ovary-

sparing procedures, particularly laparoscopic cystectomy/fenestration (36.0% vs. 15.6%; $p = 0.0003$). Detorsion was predominantly laparoscopic and showed no significant difference between groups (PD 19.9% vs. Non-PD 21.7%; $p = 0.808$), while open detorsion was extremely rare in both settings (~1%). Oophorectomy revealed a marked divergence: laparoscopic oophorectomy rates were similar (11.4% vs. 7.4%; $p = 0.270$), but open oophorectomy was significantly more common in PD (26.2% vs. 4.2%; $p < 0.0001$). Oophoropexy was rare overall (~2%).

Interpretation: Non-PD departments show a clear preference for laparoscopic, ovary-sparing approaches. In contrast, PD departments, likely confronted with younger and more complex cases, exhibit substantially higher open oophorectomy rates, which suggests either delayed presentation or more severe intraoperative findings.

Supplement Table 2 Surgical procedures by hospital type (UM vs. Non-UM)

Type of treatment / procedure	All departments (n=330)	UM (n=67)	Non-UM (n=263)	p-value
**Detorsion (lap)**	69 (20.8%)	17 (25.4%)	52 (19.8%)	0.370
**Detorsion (open / non specified)**	4 (1.2%)	0 (0.0%)	4 (1.5%)	0.589
**Cyst fenestration / cystectomy (lap)**	90 (27.2%)	14 (20.9%)	76 (28.9%)	0.296
**Cyst excision/incision (open / non specified)**	6 (1.8%)	2 (3.0%)	3 (1.1%)	0.103
**Oophorectomy (lap)**	30 (9.1%)	3 (4.5%)	27 (10.3%)	0.256
**Oophorectomy (open / non specified)**	45 (13.6%)	19 (28.4%)	26 (9.9%)	0.001
**Parovarian cyst excision (lap)**	20 (6.0%)	3 (4.5%)	17 (6.5%)	0.782
**Parovarian cyst excision (open / non specified)**	1 (0.3%)	0 (0.0%)	1 (0.4%)	1.000
**Ovariopexy (lap)**	8 (2.4%)	2 (3.0%)	6 (2.3%)	0.668
**Wedge excision / biopsy (lap)**	28 (8.5%)	2 (3.0%)	26 (9.9%)	0.100
**Wedge excision / biopsy (open / non specified)**	3 (0.9%)	1 (1.5%)	2 (0.8%)	0.494
**Miscellaneous / non specified**	19 (5.7%)	1 (1.5%)	18 (6.8%)	0.151
**Single-port laparoscopy**	3 (0.9%)	2 (3.0%)	1 (0.4%)	0.107
**Adhesiolysis (lap)**	3 (0.9%)	0 (0.0%)	3 (1.1%)	1.000
**Adhesiolysis (open / non specified)**	2 (0.6%)	1 (1.5%)	1 (0.4%)	0.365

Supplement Table 2. Surgical procedures by hospital type (UM vs. Non-UM): Laparoscopic detorsion was slightly more frequent in UM

compared to Non-UM hospitals (25.4% vs. 19.8%; p = 0.370), while open detorsion occurred only in Non-UM (1.5%). Cystectomy/fenestration (lap) was more often performed in Non-UM (28.9% vs. 20.9%; p = 0.296), though without statistical significance. For oophorectomy, a clear divergence emerged: laparoscopic oophorectomy was somewhat less frequent in UM (4.5% vs. 10.3%; p = 0.256), whereas open oophorectomy was markedly higher in UM (28.4% vs. 9.9%; p = 0.0011). Wedge excision (lap) tended to be more frequent in Non-UM (9.9% vs. 3.0%; p = 0.100).

Interpretation: UM hospitals appear to treat a higher proportion of younger and potentially more complex cases, reflected by their significantly elevated open oophorectomy rates. Non-UM hospitals, in contrast, performed more laparoscopic, ovary-sparing procedures such as cystectomy/fenestration and wedge excision, though these differences did not reach statistical significance.

Supplement Table 3 Common distribution of the procedures

Combination	Frequency	Percentage
**Detorsion + Cystectomy**	**23**	**28.4%**
**Cystectomy + Parovarian cyst excision**	**6**	**7.4%**
**Detorsion + Wedge excision**	**10**	**12.3%**
**Detorsion only**	**46**	**13.9%**
**Cystectomy only**	**30**	**9.1%**
**Oophorectomy only**	**45**	**13.6%**
**Ovariopexy only**	**8**	**2.4%**
**Multiple minor combinations**	**20**	**6.1%**
**Other (n<2 each)**	**142**	**16.8%**

Supplement Table 3. Totally 330 procedures and 81 OPS combinations were identified. Nearly half of all combination were captured by (Detorsion + Cystectomy 28.4%; Detorsion + Wedge 12.3%; Cystectomy + Parovarian cyst excision 7.4%). Simple single procedures (Detorsion only, Oophorectomy only, Cystectomy only) accounted for >35% of cases. Numerous rare combinations (<2 cases each) together represented only 16.8% of the cohort. Interpretation: The procedural spectrum was diverse but dominated by a few standard combinations.
